# Mergeomics: a web server for identifying pathological pathways, networks, and key regulators via multidimensional data integration

**DOI:** 10.1186/s12864-016-3057-8

**Published:** 2016-09-09

**Authors:** Douglas Arneson, Anindya Bhattacharya, Le Shu, Ville-Petteri Mäkinen, Xia Yang

**Affiliations:** 1 Department of Integrative Biology and Physiology, University of California, Los Angeles, CA 90095 USA; 2 South Australian Health and Medical Research Institute, Adelaide, Australia; 3 School of Biological Sciences, University of Adelaide, Adelaide, Australia; 4 Institute of Health Sciences, University of Oulu, Oulu, Finland

**Keywords:** Multidimensional data integration, Omics integration, Web server, Pathway meta-analysis, Network meta-analysis, Disease network, Key driver, GWAS, EWAS, TWAS

## Abstract

**Background:**

Human diseases are commonly the result of multidimensional changes at molecular, cellular, and systemic levels. Recent advances in genomic technologies have enabled an outpour of omics datasets that capture these changes. However, separate analyses of these various data only provide fragmented understanding and do not capture the holistic view of disease mechanisms. To meet the urgent needs for tools that effectively integrate multiple types of omics data to derive biological insights, we have developed Mergeomics, a computational pipeline that integrates multidimensional disease association data with functional genomics and molecular networks to retrieve biological pathways, gene networks, and central regulators critical for disease development.

**Results:**

To make the Mergeomics pipeline available to a wider research community, we have implemented an online, user-friendly web server (http://mergeomics.research.idre.ucla.edu/). The web server features a modular implementation of the Mergeomics pipeline with detailed tutorials. Additionally, it provides curated genomic resources including tissue-specific expression quantitative trait loci, ENCODE functional annotations, biological pathways, and molecular networks, and offers interactive visualization of analytical results. Multiple computational tools including Marker Dependency Filtering (MDF), Marker Set Enrichment Analysis (MSEA), Meta-MSEA, and Weighted Key Driver Analysis (wKDA) can be used separately or in flexible combinations. User-defined summary-level genomic association datasets (e.g., genetic, transcriptomic, epigenomic) related to a particular disease or phenotype can be uploaded and computed real-time to yield biologically interpretable results, which can be viewed online and downloaded for later use.

**Conclusions:**

Our Mergeomics web server offers researchers flexible and user-friendly tools to facilitate integration of multidimensional data into holistic views of disease mechanisms in the form of tissue-specific key regulators, biological pathways, and gene networks.

**Electronic supplementary material:**

The online version of this article (doi:10.1186/s12864-016-3057-8) contains supplementary material, which is available to authorized users.

## Background

Human diseases are complex in nature and commonly involve pathological changes at multiple molecular, cellular and systemic levels [1]. The recent revolution in genomic technologies has enabled the generation of massive amounts of molecular data encompassing genetics, transcriptomics, epigenomics, metabolomics, and proteomics, which have become easily accessible in public domains and private sectors. It is increasingly recognized that analysis of individual types of data separately only reveals a fraction of the complex biology and often misses the key players driving diseases, making multidimensional big data integration an urgent need [1–3]. To date, the majority of the available data integration tools focus on individual data types or analysis steps and lack flexibility in accommodating diverse types of genomic data. Users are typically required to turn to multiple tools to bridge the gaps between wet lab data and well-founded hypothesis for future experimental exploration. Variations in performance and potential incompatibility between tools further amplify the difficulty in big data integration.

To overcome these challenges, we have developed an R package, Mergeomics (Shu et al., companion manuscript), which has the capacity to integrate summary-level disease association data, functional genomics (such as expression quantitative trait loci (eQTLs) and ENCODE annotations), biological pathways, and gene networks to identify disease-associated gene subnetworks and key regulatory genes. Examples of disease association data are genetic association studies via genome-wide association studies (GWAS) or exome sequencing, transcriptome-wide association (TWAS) via microarray or RNA sequencing studies, and epigenome-wide association studies (EWAS). Mergeomics has a streamlined workflow to incorporate multiple analysis components in modular format, allowing flexibility in accommodating a variety of data types and study designs.

In order to achieve this flexibility in data types and study design, it is not feasible to deal with these diverse data types in their native, pre-processed format. We find that the association of markers (e.g., SNPs, genes, methylation sites) to a phenotype from various data modalities is a converging point that allows us to apply our strategy across such data types as GWAS, microarray, RNA sequencing, DNA methylation, etc. In the context of the current study, we use the term “association dataset” in a broader sense by referring to the association statistics between any omics-derived markers and a phenotype. The applicability of the approach to various data types including GWAS, TWAS, and EWAS has been demonstrated in the companion paper. It is also feasible for metabolome and proteome association datasets. In addition, the analytical pipeline can be used for datasets from any species as long as the corresponding species-specific datasets and information are provided. Furthermore, our pipeline offers a meta-analysis component that can converge findings across data types and/or species in a single analysis, as demonstrated in the companion method paper.

To maximize the usability of Mergeomics, here we present a user-friendly web server with full implementation of the features in the Mergeomics R package. The web server features four analytical modules that can correct for dependencies between omics markers (MDF - Marker Dependency Filtering), identify biological pathways and networks that are enriched for disease-associated signals from a single omics study (MSEA - Marker Set Enrichment Analysis) or from two or more omics studies of the same or different data types (Meta-MSEA), and identify key network regulators or hubs of the disease-associated pathways and networks (wKDA - Weighted Key Driver Analysis). The web server is also accompanied by a detailed step-by-step tutorial and pre-processed, commonly used datasets such as functional genomics, pathways, and gene networks as resources to facilitate data integration. Users have the full flexibility to select analysis modules that best suit their study design and data types. Summary results and interactive network views are displayed online and links to detailed downloadable results are also available.

## Implementation

The Mergeomics web server is hosted within UCLA’s Hoffman2 High Performance Cluster as part of the Institute for Digital Research and Education (IDRE), and jobs are submitted using the Sun Grid Engine queuing system. The web server front-end is written in HTML, PHP and JavaScript and hosted through an Apache HTTP server, which allows users to submit jobs through web browsers (all major browsers are supported). A 400 MB file size upload restriction is imposed to ensure reasonable compute time. User files are temporarily kept under a randomly generated file name on the web server during the analysis. As soon as the compute completes, user uploaded files are deleted from the server. Submitted jobs are assigned unique alphanumeric identifiers that allow users to control access to their results. Only the job submitter will be given the job-specific links to download the result files, which are kept on the web server for 24 h to allow sufficient time to download. Users can provide an email address (optional) if they would like to receive notifications about their job status and have results emailed upon task completion. Sever traffic is monitored through the total number of hits and the number of unique hits.

## Results and discussion

### Web server overview

**Fig. 1:**
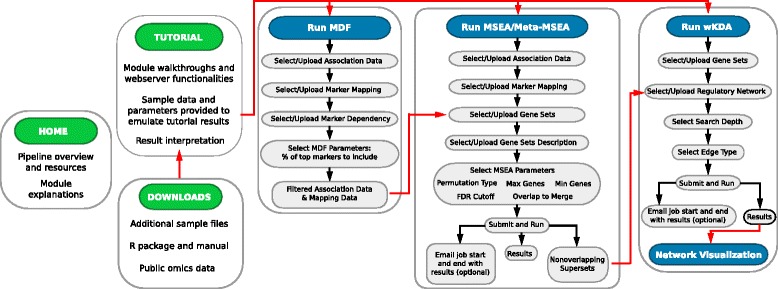
Web server pipeline overview with *red lines* indicating cross-module interactions, *green headers* indicating resource pages, and *teal headers* indicating analytical modules

As depicted in Fig. 1, the web server hosts three resource pages (HOME, TUTORIAL, and DOWNLOAD) and four analytical modules (MDF, MSEA, Meta-MSEA, and wKDA). The “HOME” page provides basic information about the Mergeomics pipeline including data types involved, overall workflow, and citation information. The “TUTORIAL” section provides step-by-step instructions for using the web server and detailed descriptions about input file format requirements, analysis parameters, and result interpretation for each analytical component. The “DOWNLOAD” section gives users access to the standalone Mergeomics R package and a wide variety of useful, publically available genomic resources applicable in the pipeline, including sample disease association studies, tissue-specific eQTL studies, ENCODE resources, knowledge-driven biochemical and signaling pathways, and data-driven gene networks (Additional file 1: Table S1). The four analytical modules are: 1) MDF, standing for Marker Dependency Filtering, which corrects for dependencies between omics markers (e.g. correcting for linkage disequilibrium or LD between genetic variants in GWAS), 2) MSEA, standing for Marker Set Enrichment Analysis, that allows users to leverage multi-omics association data, functional genomics, canonical pathways and/or data-driven gene modules to identify causal subnetworks of disease or traits; 3) Meta-MSEA, which performs pathway- or network-level meta-analysis when multiple association datasets from different studies (e.g., multiple GWAS) and/or of different data types (e.g., GWAS and EWAS) are available; 4) wKDA, standing for weighted key driver analysis, which maps disease associated genes to tissue-specific gene networks to identify potential key drivers or regulators of disease. We also provide an interactive network visualization module for visualizing select key drivers and their associated disease subnetworks.

**Fig. 2:**
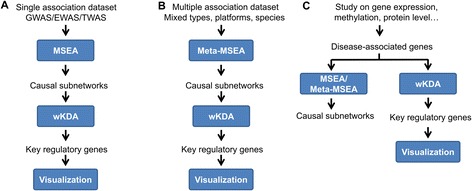
Flexibility of Mergeomics in accommodating different datasets and study designs. **a** When a single disease association dataset is available, an MSEA-wKDA-visualization flow can be utilized. **b** When multiple disease association datasets are available, use a Meta-MSEA then wKDA flow is more appropriate. **c** When selected disease-associated genes or proteins are already identified, wKDA can be directly used. MSEA could also be run if additional association datasets are available, allowing for association testing in other diseases or cross-species validation

To use the web server, users follow a streamlined workflow (Fig. 1) by setting up the parameters and selecting or uploading datasets necessary for each of the analytical components. Due to the modular design, users are allowed the flexibility to choose subsets of the analytical components according to available datasets and specific study design (Fig. 2). For example, if a single association dataset (e.g., GWAS/EWAS/TWAS) is available, users can identify the causal subnetwork and key regulatory gene of a trait or disease using the MSEA-wKDA-Visualization workflow (Fig. 2a). If multiple association datasets of either the same data type or different data types are available, the Meta-MSEA-wKDA-Visualization workflow is more appropriate (Fig. 2b). If only groups of disease-associated genes are available, the wKDA-Visualization workflow is sufficient to generate the key regulators and disease subnetworks, and users could still explore the association of the gene sets with the same disease in other organisms or other relevant disease types using MSEA or Meta-MSEA, if the corresponding association data is available (Fig. 2c). As technological advances bring about new data types, they can be effectively incorporated into the analysis framework of Mergeomics. In the following sections we explain each analytical component of the Mergeomics web server in detail.

### Marker dependency filtering (MDF)

Depending on the type of data to be analyzed in MSEA or Meta-MSEA, an additional step may be required to correct for any dependencies between the markers (e.g., LD between single nucleotide polymorphisms (SNPs) in GWAS, correlation between adjacent methylation sites, etc.). For GWAS, we provide on-the-fly pruning of SNPs based on HapMap3 LD information and user-defined LD cutoffs. For other types of markers, users need to prepare and upload the marker association files containing independent markers. If dependency among markers is not corrected, spurious results may be generated.

#### Data input

1. Association summary results between molecular markers and a disease or phenotype as described in the following MSEA section.
2. Gene-marker mapping file as described in the MSEA section.
3. Marker dependency file, which describes the pairwise relationships of the markers in the association file.

#### Parameter setting

Two parameters are required for MDF: marker dependency cutoff and the percentage of top markers to be used. For GWAS, LD cutoff defines the maximum acceptable r^2^ values when pruning the SNPs, which is done using the built-in MDPRUNE program of Mergeomics pipeline. The MDPRUNE program is also downloadable from the web server and can be run independently for marker pruning. The percentage of top marker parameter is introduced to increase signal to noise ratio since more noise tends to be present towards the weaker association spectrum [4]. Default parameters yield optimal performance for GWAS based on simulation.

#### Result interpretation

The output of MDF contains the marker-disease association results only for independent markers after pruning, which can be directly used in the downstream MSEA or meta-MSEA analysis.

### Marker-set enrichment analysis (MSEA)

MSEA proceeds in the following order. Step 1: Upload marker-level association data. Only summary level association results featuring marker IDs and association –log10 *p*-values are needed. Step 2: Map marker-level association *p*-values to genes based on available genomic mapping. Step 3: Assign *p*-values to knowledge-driven or data-driven gene sets in the form of pathways of gene subnetwork modules. Step 4: Perform enrichment analysis and output gene sets significantly enriched for disease-associated markers. Step 5: Merge top significant gene sets into non-overlapping supersets to reduce redundancy.

#### Data preparation

MSEA requires three different types of data as input, and our web server provides a number of public datasets and allows users to upload datasets of their interest.

1. Marker-disease association summary results including Marker IDs and –log10 transformed association *p*-values (Fig. 3a). Marker types can be SNPs, methylation loci, transcripts, proteins, metabolites, etc. (sample datasets provided in the web server). Non-independent relationship between markers should be corrected if possible (using MDF described above).

**Fig. 3:**
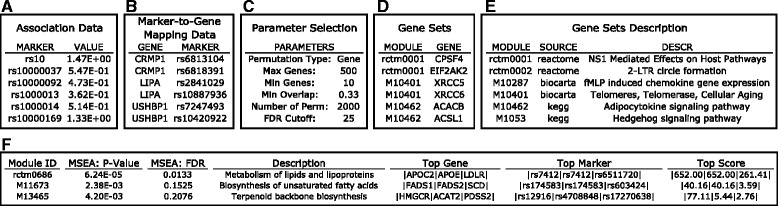
Screenshots showing a step-by-step overview of the MSEA module using sample data on the web server. **a** Association data format. **b** Marker mapping data format. **c** Parameters. **d** Gene sets format. **e** Gene sets descriptions. **f** Example result summary table containing the IDs and descriptions of the gene sets tested to be associated with the disease, the *p* - values, FDR values, top genes and markers with the strongest disease association within each gene set, and the strongest association scores in the form of –log10 *p* - values
2. Gene-marker mapping file that links genomic markers to genes (Fig. 3b). For GWAS, the most commonly used mapping is based on genomic distance (e.g., 10 kb, 20 kb, 50 kb), which is provided on the web server. Such distance mapping could be applied to methylation sites in EWAS as well. A data-driven function-based mapping is more preferred if available. To facilitate this aspect, we provide curated publically available functional genomics resources from ENCODE [5] and various tissue-specific eQTLs from numerous studies including GTEx [6] and Muther [7] (Additional file 1: Table S1). Users can upload their own mapping files between markers and genes.
3. Functionally related gene sets defined based on various criteria (Fig. 3d), such as co-regulation, shared response, co-localization on chromosomes, or participants of specific biological processes or signaling pathways. Typical sources of gene sets includes canonical pathways such as Reactome [8] and KEGG [9], coexpression modules constructed using algorithms like weighted coexpression gene networks analysis (WGCNA) [10], and gene signatures from gene expression profiling studies. We provide curated functional gene sets from these sources on our web server (Additional file 1: Table S1), but also allow users to upload their own gene sets. To better annotate the gene sets in MSEA output, the user could optionally provide a description file for the gene sets to specify the source of the gene set and a detailed description of the functional information used to group genes (Fig. 3e).

#### Parameter setting

The core parameter of MSEA is the permutation type for assessing null distribution. Either marker-level permutation or gene-level permutation can be selected. Gene-level permutation is set as default as it has higher true positive rate, low false positive rate, and low sensitivity to variation in other parameters (Shu et al., companion manuscript). However, users may use the more sensitive marker-level permutation for suggestive signals. Additional parameters include the maximum and minimum number of genes in gene sets, false discovery rate (FDR) cutoff to determine significant signals, and merging criteria for overlapping gene sets (Fig. 3c; detailed in online tutorial).

#### Result interpretation

On the result webpage, a summary table for the top disease-associated gene sets at a user-defined cutoff is generated for quick reference of the MSEA results (Fig. 3f). This summary table reports the gene set ID, disease association enrichment *p* - value of the gene set, FDR, gene set description, top 3 genes and top 3 markers in each gene set that contribute to the overall enrichment signal, and the association *p* - values (in –log10 format) of the markers. The detailed results of MSEA are provided in four downloadable tables: 1) “MSEA_modules_pval.txt”: a list of gene sets ranked by enrichment *p* -values and false discovery rate (FDR). 2) “MSEA_modules_full_results.txt”: detailed enrichment results including enrichment *p* -values, number of mapped genes and markers for each gene set, and the density ratio which gives the ratio of unique markers mapped to gene set divided by the total markers in the analysis. 3) “MSEA_top_modules_details.txt”: detailed list of genes from top gene sets (default: FDR < 25 %), accompanied by the total number of markers, top markers and the highest –log10 transformed association *p* -value for each gene. Users could use this output to track the origin of enrichment signals as well as to identify potentially important genes and markers in each of the significant gene sets. 4) “MSEA_genes_details.txt”: stores gene-related MSEA results, including enrichment score, number of markers mapped to each gene, and markers with top association values for each gene.

### Meta-MSEA

When multiple disease association datasets are available, Meta-MSEA can be used to conduct meta-analysis at the pathway or network level. This function allows users to achieve maximal power by combining results from independent association studies of different ethnicities, platforms or even species, while avoiding the technical difficulties when performing meta-analysis directly on the marker-level association data [11]. To run meta-MSEA, users simply need to navigate to the Meta-MSEA tab, and upload multiple datasets following the same workflow as previously described for MSEA to generate results for individual datasets as well as the pathway/network-level meta-analysis results. The result files produced by Meta-MSEA follow the same layout as MSEA.

### Weighted key driver analysis (wKDA)

wKDA aims to pinpoint key regulator genes or key drivers (KDs) of the disease related gene sets from MSEA or meta-MSEA using gene network topology and edge weight information. Specifically, wKDA first screens the network for candidate hub genes. Then the disease gene sets are overlaid onto the subnetworks of the candidate hubs to identify KDs whose neighbors are enriched with disease genes.

#### Data preparation

**Fig. 4:**
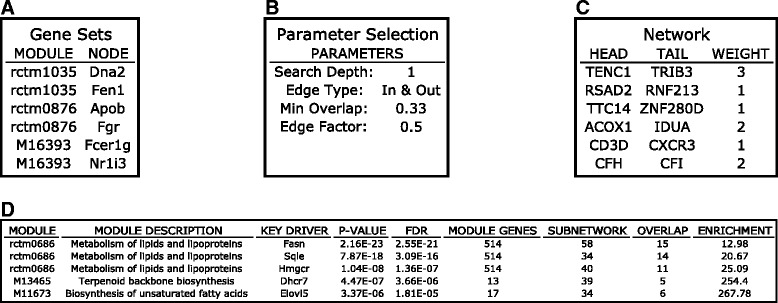
Screenshots showing a step-by-step overview of the wKDA module using sample data on the web server. **a** Gene set format. **b** Parameters. **c** Network format. **d** Example result summary table containing the IDs of the disease associated gene sets, their KDs, *p* - values and FDRs of the KDs, gene counts of KD subnetworks, and fold enrichment of the KD subnetworks for disease genes

Two types of files are required for wKDA: 1) disease-associated gene sets (Fig. 4a) and 2) molecular networks (Fig. 4c). wKDA can be run as either the continuing step of MSEA or meta-MSEA or as an independent step (Fig. 2). If the user elects to continue wKDA from MSEA or meta-MSEA, then the enriched gene sets from these analyses will be used as the disease-associated gene sets. If the user elects to run wKDA as a separate module, they must upload their own gene sets to the web server or they can use the pre-loaded sample gene set for testing. With regards to molecular networks, wKDA supports a wide range of directed and undirected regulatory networks. wKDA is designed to utilize edge weight information in gene networks, which could be connection strength or reliability measures. If no edge weight information is available, wKDA can also operate by considering equal weights to all edges. The web server provides a collection of tissue-specific Bayesian networks previously constructed in human and mouse studies (Additional file 1: Table S1). There are also a large number of publicly available network resources, such as protein-protein interactions (PPI) [12], BioGRID [13], GeneMANIA [14] and GIANT [15], which could be used in wKDA.

#### Parameter setting

Core wKDA parameters include 1) search depth, which specifies the number of layers to expand in the network when determining the neighboring genes of candidate KDs for enrichment assessment, and 2) edge directionality, which specifies whether to neglect edge directionality (incoming and outgoing) or require the candidate hubs to be upstream of neighbor genes (only outgoing) for networks that carry directionality information (Fig. 4b). Additional parameters are described in detail in the online tutorial.

#### Result interpretation

Summary results of wKDA will be displayed on the webpage (Fig. 4d), which reports top 5 KDs for each disease gene set along with the statistics. Users could also download four detailed results files: 1) “wKDA_kd_*p*values.txt”, a summary table of all KDs ranked by *p* -values and FDR; 2) “wKDA_kd_full_results.txt”, providing detailed statistics on all KDs identified; 3) “wKDA_kd_tophits.txt”, *p*-value summary table for only the top KDs for each disease gene set. 4) “wKDA_hub_structure.txt”, specifying hub-cohub relationship. The co-hub structure is useful to group KDs with highly overlapping network topology, and to retrieve list of independent KDs for more efficient prioritization. Additionally, wKDA provides Cytoscape-ready files that can be used in Cytoscape [16] for a more customized visualization than the included web-based network visualization module.

#### Network visualization

**Fig. 5:**
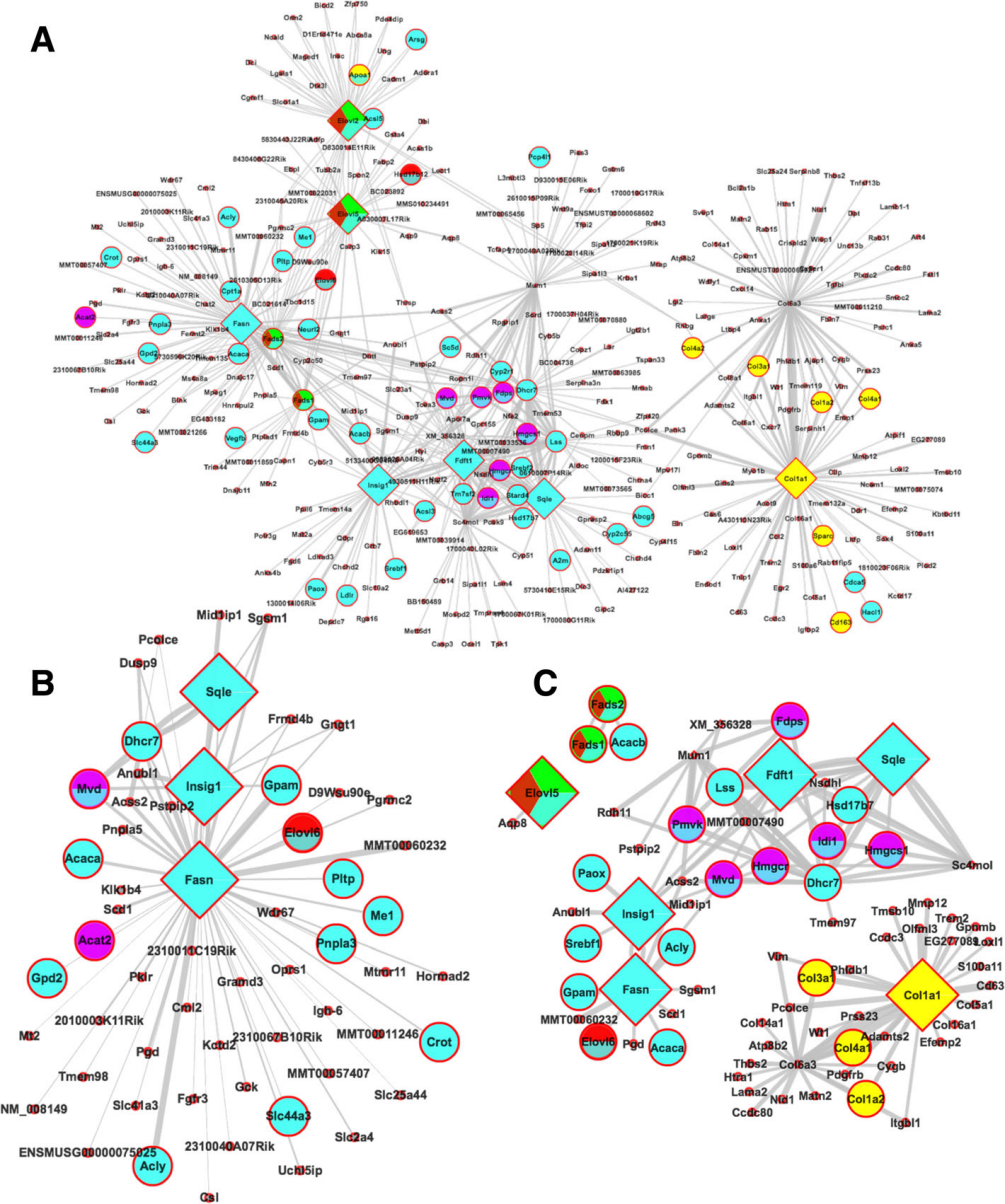
Screenshots of the network visualization module output generated by the default sample files in the wKDA module. **a** Overview of a network comprised of top KDs for LDL-associated gene sets, with 5 KDs for each gene set displayed. KDs are depicted as diamond nodes and node colors indicating gene set membership. **b** Focused subnetwork view of key driver of interest *Fasn* when filtering the network in (**a**) by submitting a “Fasn” query or right clicking the node. **c** The network can be filtered to display only nodes with edge weights higher than a certain threshold: 4.0 in this case

Our web server provides a convenient module to allow users visualize top KDs and subnetworks using Cytoscape Web v0.8 [17]. The top 5 KDs for each disease gene set from wKDA will be automatically visualized, as exemplified in Fig. 5a. The visualization is interactive so that users can make real-time changes such as zooming in on a node of interest by only considering that particular subnetwork (Fig. 5b) or by filtering a subnetwork based on the edge weight information (Fig. 5c), as detailed in the tutorial page.

### Application example

As an illustration of our web server’s workflow and analysis results, we applied the Mergeomics web server to a publically available low-density lipoprotein (LDL) GWAS dataset from the GLGC consortium [18]. All files mentioned within this section are provided as example files on the web server. To correct for LD between SNPs, we used the MDF analysis module and the following input files: the GLGC LDL GWAS summary statistics (SNPs, −log10 *p* - values), SNP-gene mapping based on 50 kb chromosomal distance, and the Hapmap CEU LD file containing SNPs with *r*^2^ > 0.7 as the Marker Dependency file. We also filtered the GWAS loci by only consider the top 50 % SNPs ranked by *p* - values to reduce random noise from the weaker association spectrum.

After correction for marker-dependency using MDF, the resulting association and mapping files were used directly as input for MSEA (Fig. 3a-b) using gene permutation and default setting for the other parameters (Fig. 3c), to test for enrichment for canonical pathways collected from KEGG, Biocarta and Reactome databases (Fig. 3d-e). Upon completion of MSEA, a summary table was produced which details the top pathways ranked by FDR along with descriptions of the pathways and top associated genes and SNPs in each pathway. As exemplified in Fig. 3f, “Metabolism of lipids and lipoproteins”, “biosynthesis of unsaturated fatty acids”, and “terepenoid backbone biosynthesis” were three of the top pathways identified among others. *APOC2*, *APOE*, and *LDLR* were listed as the top associated genes in the “metabolism of lipids and lipoproteins” pathway, and their corresponding SNPs were also provided in the summary table. Links to detailed result tables were also displayed for file download. Furthermore, these top pathways were checked for overlaps and merged if significant overlaps in gene membership between pathways were identified. A summary table of the merged pathways was also displayed (not shown).

To identify potential KDs and subnetworks for the LDL-associated pathways, the merged pathways were used directly as input for wKDA (Fig. 4a). wKDA was run using default parameters (Fig. 4b) and a liver Bayesian network (Fig. 4c). Upon completion, a summary table is produced (Fig. 4d) which lists the top 5 KDs for each merged module and information about their local subnetwork structure. For example, *Fasn* was a KD for the Metabolism of Lipids and Lipoproteins pathway. Links to detailed result tables were also displayed for file download.

The wKDA results can be viewed directly using the interactive visualization feature, which by default illustrates the top 5 KDs for each gene set and their local subnetworks with disease genes highlighted (Fig. 5a). The networks can be filtered by selecting a particular KD of interest to focus on (Fig. 5b) or by removing edges below an edge weight cutoff to focus on high confidence network connections (Fig. 5c). To facilitate further customization of network views, Cytoscape-ready files can be downloaded for external visualization.

## Conclusions

We have implemented the Mergeomics pipeline as a user-friendly, publicly available web server that can facilitate multidimensional omics data integration to expedite novel discoveries. The web server also pre-populates a wide range of publically available data sources. Users can apply the pipeline to their own data in conjunction with any preloaded data to identify disease-associated pathways, gene networks, and key regulators. The web server includes step-by-step tutorials, examples and visualization tools in a web-based platform. The flexibility of the web server to accommodate various omics data types and to conduct pathway and network-level meta-analysis of multiple studies of different design will boost our ability to integrate big data.

## Additional file

Additional file 1: Table S1. List of public datasets available for access in the Mergeomics web server. (DOCX 128 kb)

## Abbreviations

ENCODE: ENCylopedia of DNA Elements
eQTLs: Expression quantitative trait loci
EWAS: Epigenome wide association study
FDR: False discovery rate
GWAS: Genome wide association study
HTTP: Hypertext transfer protocol
KD: Key driver
KEGG: Kyoto encyclopedia of genes and genomes
LD: Linkage disequalibrium
LDL: Low-density lipoprotein
MDF: Marker dependency filtering
Meta-MSEA: Meta marker set enrichment analysis
MSEA: Marker set enrichment analysis
PHP: Hypertext preprocessor
PPI: Protein-protein interaction
SNP: Single nucleotide polymorphism
TWAS: Transcritome wide association study
WGCNA: Weighted gene coexpression network analysis
wKDA: Weighted key driver analysis
