# Supplementary material for: Mergeomics: a web server for identifying pathological pathways, networks, and key regulators via multidimensional data integration
Source: BMC Genomics. 2016 Sep 9;17(1):722. doi: 10.1186/s12864-016-3057-8 (PMC5016927; doi:10.1186/s12864-016-3057-8)
Supplement: Additional file 1: Table S1. — List of public datasets available for access in the Mergeomics web server. (DOCX 128 kb) [file 12864_2016_3057_MOESM1_ESM.docx]

**Supplemental Table 1. Data resources available on Mergeomics web server.**

| **Data Category** | **Data Category Description** | **Preloaded Sample File Name** | **Sample File Description** | **Sample Data References** |
| --- | --- | --- | --- | --- |
| Disease association data | Marker to trait association | glgc.tc | Total cholesterol GWAS | [[1](#_ENREF_1)] |
|  |  | glgc.tg | Triglycerid GWAS |  |
|  |  | glgc.ldl | LDL GWAS |  |
|  |  | glgc.hdl | HDL GWAS |  |
|  |  | cardiogram_c4d.cad | Coronary artery disease GWAS | [[2](#_ENREF_2)] |
|  |  | diagram.t2d | Type 2 diabetes GWAS | [[3](#_ENREF_3)] |
|  |  | magic.fastingglucose | Fasting glucose GWAS | [[4](#_ENREF_4)] |
| Marker mapping Data | Marker to gene mapping | esnp.adipose | Adipose eQTLs | [[5-8](#_ENREF_5)] |
|  |  | esnp.blood | Blood eQTLs | [[5](#_ENREF_5), [7](#_ENREF_7), [9](#_ENREF_9)] |
|  |  | esnp.brain | Brain eQTLs | [[10-13](#_ENREF_10)] |
|  |  | esnp.liver | Liver eQTLs | [[5](#_ENREF_5), [8](#_ENREF_8), [14](#_ENREF_14)] |
|  |  | esnp.muscle_skeletal | Skeletal muscle eQTLS | [[5](#_ENREF_5)] |
|  |  | gene2loci.010kb | Human SNP to gene mapping based on a chromosomal distance of 10kb |  |
|  |  | gene2loci.020kb | Human SNP to gene mapping based on a chromosomal distance of 20kb |  |
|  |  | gene2loci.050kb | Human SNP to gene mapping based on a chromosomal distance of 50kb |  |
|  |  | gene2loci.regulome | Human SNP to gene mapping based on RegulomeDB (ENCODE) | [[15](#_ENREF_15)] |
| Gene Sets | Collections of pre-defined sets of genes that are functionally related | Canonical pathways | Pathways collected from KEGG, REACTOME and Biocarta | [[16](#_ENREF_16), [17](#_ENREF_17)] |
|  |  | Co-expression modules | Derived from coexpression networks by applying WGCNA on gene expression data | [[7](#_ENREF_7), [8](#_ENREF_8), [10-14](#_ENREF_10), [18](#_ENREF_18)] |
| Gene regulatory networks | Network edges from pre-defined gene networks | adipose | Adipose Bayesian networks | [[7](#_ENREF_7), [10-14](#_ENREF_10)] |
|  |  | blood | Blood Bayesian networks | [[7](#_ENREF_7)] |
|  |  | brain | Brain Bayesian networks | [[10-13](#_ENREF_10)] |
|  |  | liver | Liver Bayesian networks | [[10-14](#_ENREF_10)] |
|  |  | muscle | Muscle Bayesian networks | [[10-14](#_ENREF_10)] |
|  |  | PPI | Protein-protein interaction network | [[19](#_ENREF_19)] |

**References**

1. Global Lipids Genetics C, Willer CJ, Schmidt EM, Sengupta S, Peloso GM, Gustafsson S, Kanoni S, Ganna A, Chen J, Buchkovich ML *et al*: **Discovery and refinement of loci associated with lipid levels**. *Nature genetics* 2013, **45**(11):1274-1283.

2. Nikpay M, Goel A, Won HH, Hall LM, Willenborg C, Kanoni S, Saleheen D, Kyriakou T, Nelson CP, Hopewell JC *et al*: **A comprehensive 1,000 Genomes-based genome-wide association meta-analysis of coronary artery disease**. *Nature genetics* 2015, **47**(10):1121-1130.

3. Mahajan A, Go MJ, Zhang WH, Below JE, Gaulton KJ, Ferreira T, Horikoshi M, Johnson AD, Ng MCY, Prokopenko I *et al*: **Genome-wide trans-ancestry meta-analysis provides insight into the genetic architecture of type 2 diabetes susceptibility**. *Nature genetics* 2014, **46**(3):234-+.

4. Dupuis J, Langenberg C, Prokopenko I, Saxena R, Soranzo N, Jackson AU, Wheeler E, Glazer NL, Bouatia-Naji N, Gloyn AL *et al*: **New genetic loci implicated in fasting glucose homeostasis and their impact on type 2 diabetes risk**. *Nature genetics* 2010, **42**(2):105-116.

5. Carithers LJ, Moore HM: **The Genotype-Tissue Expression (GTEx) Project**. *Biopreservation and biobanking* 2015, **13**(5):307-308.

6. Nica AC, Parts L, Glass D, Nisbet J, Barrett A, Sekowska M, Travers M, Potter S, Grundberg E, Small K *et al*: **The architecture of gene regulatory variation across multiple human tissues: the MuTHER study**. *PLoS genetics* 2011, **7**(2):e1002003.

7. Emilsson V, Thorleifsson G, Zhang B, Leonardson AS, Zink F, Zhu J, Carlson S, Helgason A, Walters GB, Gunnarsdottir S *et al*: **Genetics of gene expression and its effect on disease**. *Nature* 2008, **452**(7186):423-428.

8. Greenawalt DM, Dobrin R, Chudin E, Hatoum IJ, Suver C, Beaulaurier J, Zhang B, Castro V, Zhu J, Sieberts SK *et al*: **A survey of the genetics of stomach, liver, and adipose gene expression from a morbidly obese cohort**. *Genome research* 2011, **21**(7):1008-1016.

9. Fehrmann RSN, Jansen RC, Veldink JH, Westra HJ, Arends D, Bonder MJ, Fu JY, Deelen P, Groen HJM, Smolonska A *et al*: **Trans-eQTLs Reveal That Independent Genetic Variants Associated with a Complex Phenotype Converge on Intermediate Genes, with a Major Role for the HLA**. *PLoS genetics* 2011, **7**(8).

10. Derry JM, Zhong H, Molony C, MacNeil D, Guhathakurta D, Zhang B, Mudgett J, Small K, El Fertak L, Guimond A *et al*: **Identification of genes and networks driving cardiovascular and metabolic phenotypes in a mouse F2 intercross**. *PloS one* 2010, **5**(12):e14319.

11. Wang SS, Schadt EE, Wang H, Wang X, Ingram-Drake L, Shi W, Drake TA, Lusis AJ: **Identification of pathways for atherosclerosis in mice: integration of quantitative trait locus analysis and global gene expression data**. *Circulation research* 2007, **101**(3):e11-30.

12. Yang X, Schadt EE, Wang S, Wang H, Arnold AP, Ingram-Drake L, Drake TA, Lusis AJ: **Tissue-specific expression and regulation of sexually dimorphic genes in mice**. *Genome research* 2006, **16**(8):995-1004.

13. Tu Z, Keller MP, Zhang C, Rabaglia ME, Greenawalt DM, Yang X, Wang IM, Dai H, Bruss MD, Lum PY *et al*: **Integrative analysis of a cross-loci regulation network identifies App as a gene regulating insulin secretion from pancreatic islets**. *PLoS genetics* 2012, **8**(12):e1003107.

14. Schadt EE, Molony C, Chudin E, Hao K, Yang X, Lum PY, Kasarskis A, Zhang B, Wang S, Suver C *et al*: **Mapping the genetic architecture of gene expression in human liver**. *PLoS biology* 2008, **6**(5):e107.

15. Boyle AP, Hong EL, Hariharan M, Cheng Y, Schaub MA, Kasowski M, Karczewski KJ, Park J, Hitz BC, Weng S: **Annotation of functional variation in personal genomes using RegulomeDB**. *Genome research* 2012, **22**(9):1790-1797.

16. Ogata H, Goto S, Sato K, Fujibuchi W, Bono H, Kanehisa M: **KEGG: Kyoto Encyclopedia of Genes and Genomes**. *Nucleic Acids Res* 1999, **27**(1):29-34.

17. Croft D, Mundo AF, Haw R, Milacic M, Weiser J, Wu G, Caudy M, Garapati P, Gillespie M, Kamdar MR *et al*: **The Reactome pathway knowledgebase**. *Nucleic Acids Res* 2014, **42**(Database issue):D472-477.

18. Erbilgin A, Civelek M, Romanoski CE, Pan C, Hagopian R, Berliner JA, Lusis AJ: **Identification of CAD candidate genes in GWAS loci and their expression in vascular cells**. *Journal of Lipid Research* 2013, **54**(7):1894-1905.

19. Rossin EJ, Lage K, Raychaudhuri S, Xavier RJ, Tatar D, Benita Y, International Inflammatory Bowel Disease Genetics C, Cotsapas C, Daly MJ: **Proteins encoded in genomic regions associated with immune-mediated disease physically interact and suggest underlying biology**. *PLoS genetics* 2011, **7**(1):e1001273.
